# Effect of Sensor Head Orientation on the Accuracy of Magnetic Defect Detection in Steel-Cord Conveyor Belts

**DOI:** 10.3390/s25237364

**Published:** 2025-12-03

**Authors:** Aleksandra Rzeszowska, Ryszard Błażej

**Affiliations:** Faculty of Geoengineering, Mining and Geology, Wroclaw University of Science and Technology, Na Grobli 15 St., 50-421 Wrocław, Poland; aleksandra.rzeszowska@pwr.edu.pl

**Keywords:** conveyor belt diagnostics, magnetic method, measurement head orientation, intelligent monitoring systems, geometric errors, inductive sensors, NDT

## Abstract

This study analyses how the orientation of the measurement head in a magnetic diagnostic system affects the parameters of magnetic signals recorded during steel-cord conveyor belt inspection. The experiments were conducted on a laboratory test stand using a reference belt with artificial defects at two belt speeds and several sensitivity thresholds. Three types of head rotation were analyzed: longitudinal (OX), transverse (OY), and planar (OZ). For each configuration, a set of geometric signal parameters was calculated, including length, width, orientation, eccentricity, and solidity. The results showed that rotation about the OX axis caused the greatest geometric distortions (increased orientation_deg and eccentricity). Rotation about the OY axis produced amplitude asymmetry and changes in solidity (circularity), while rotation about the OZ axis resulted in twisting and displacement of the signal centroid. The total area (area_mm2) remained stable, confirming the geometric nature of the observed changes. Even small head deviations (5–10°) may introduce significant interpretation errors. Therefore, the application of geometric calibration and orientation compensation algorithms is recommended to improve the online diagnostic accuracy of the measurement system.

## 1. Introduction

Conveyor belts are a primary means of bulk material transport in open-pit and underground mining, power generation, transhipment ports, and the cement and chemical industries. They ensure process continuity at low unit costs and high energy efficiency over long distances [1,2]. With the increasing length of conveyor lines (up to several tens of km) and growing material throughput, condition-based maintenance (CBM) and the modernization of drive systems (VFDs, speed control) have become increasingly important, as confirmed by recent reviews of energy-efficient conveyor solutions [3,4].

**Figure 1:**
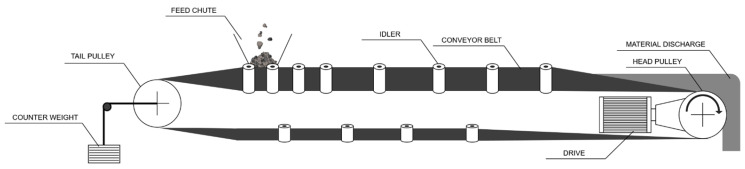
Schematic diagram of a belt conveyor.

A typical belt conveyor consists of a conveyor belt, drive unit(s) (motor–gearbox–drive pulley), return–tensioning station, sets of carrying and return idlers, cleaning systems, and control equipment. The schematic structure of a belt conveyor is shown in Figure 1.

**Figure 2:**
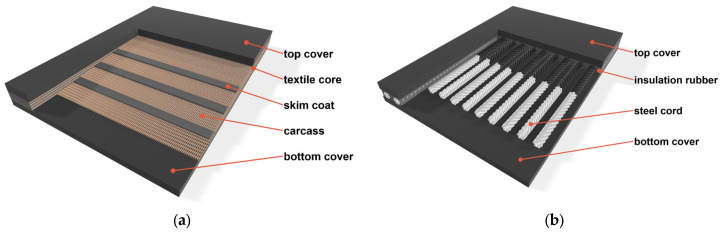
Construction of conveyor belts: (**a**) textile-cord type, (**b**) steel-cord type.

The conveyor belt is a continuous closed loop whose function is to transport material [5]. Belts are manufactured in sections, with their length limited by transport weight and reel diameter. Depending on the nominal belt strength (and thus weight), belts are produced in lengths of 200–500 m for steel-cord belts (St) and up to 800 m for textile-cord belts. Conveyor belts are divided according to core construction into the following:

- Textile-Cord Belts (EP/NN)—The carcass consists of fabric plies (polyester/nylon or, less commonly, nylon/nylon). They are lighter, more flexible, and widely used in shorter conveyors under moderate tension. In the industrial literature and operational studies, they are described as the preferred solution for typical process industries and shorter routes. A schematic of a textile-cord belt is shown in Figure 2a.
- Steel-Cord Belts (ST)—The core consists of parallel steel cords embedded in rubber, covered with upper and lower rubber covers. These belts exhibit higher tensile strength and lower elongation, making them the standard for long and/or heavily loaded conveyors [2]. The construction of a steel-cord belt is shown in Figure 2b.

For users, the practical difference is straightforward: textile belts are cheaper and easier to install, but their lower strength limits permissible tension and length, whereas steel-cord belts require more precise splicing but provide higher strength (ST classes) and dimensional stability over long distances [6].

Since belts are manufactured in several-hundred-meter sections, the loop is formed by joining the ends using an appropriate splice type—for ST belts only hot-vulcanized joints are allowed, while textile belts may be joined by hot vulcanization, cold vulcanization (adhesive), or mechanical fasteners [7,8]. Each conveyor loop must contain at least one splice, although in practice there are usually several (sometimes dozens). From a reliability perspective, splices are the weakest element of the loop, and their number significantly affects the overall system reliability [9,10].

**Figure 3:**
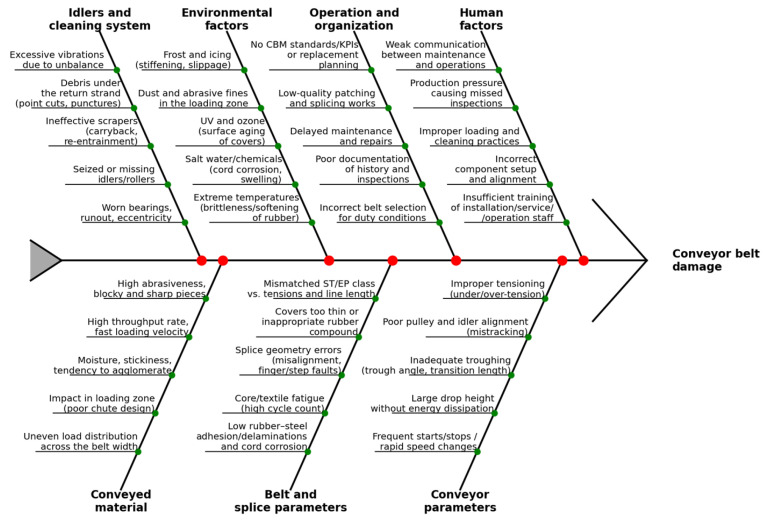
Sources of conveyor belt damage—Ishikawa diagram.

In ST belts, the main risks involve cord breakage, corrosion-induced weakening, and degradation of rubber–steel adhesion; in textile belts, fatigue and cuts in the plies, as well as cover wear, dominate. Degradation results from a combination of material factors (rubber composition, core design), operational factors (impact, particle size, humidity), system elements (idler condition, runout, misalignment), and measurement/monitoring conditions (sensor geometry, lift-off, magnetic saturation). A convenient way to organize these relationships is the Ishikawa diagram (Figure 3), which summarizes causes → damage mechanisms → symptoms → detection methods [2,11,12,13,14,15,16].

High-performance conveyor systems constitute critical infrastructure in mining and industry, and their reliability depends largely on the condition of the steel cords and the quality of splices. In recent years, there has been a clear shift from visual inspections toward non-destructive testing (NDT) and intelligent sensor-based monitoring, enabling the transition from time-based to condition-based maintenance (CBM). This allows cost reduction through scheduled overhauls, belt refurbishment, and optimization of belt life cycles [17,18].

Among NDT methods for steel-cord belt diagnostics, the Magnetic Flux Leakage (MFL) technique holds a dominant position, as it visualizes field disturbances at cord discontinuities (breaks, cross-section loss, corrosion). Current technical reviews indicate that the MFL signal’s sensitivity and shape depend strongly on (i) sensor lift-off, (ii) magnetization direction and intensity, (iii) relative velocity, and (iv) sensor orientation with respect to the tested object [17,19,20,21]. Variations in these parameters modify the signal amplitude, symmetry, and position.

**Figure 4:**
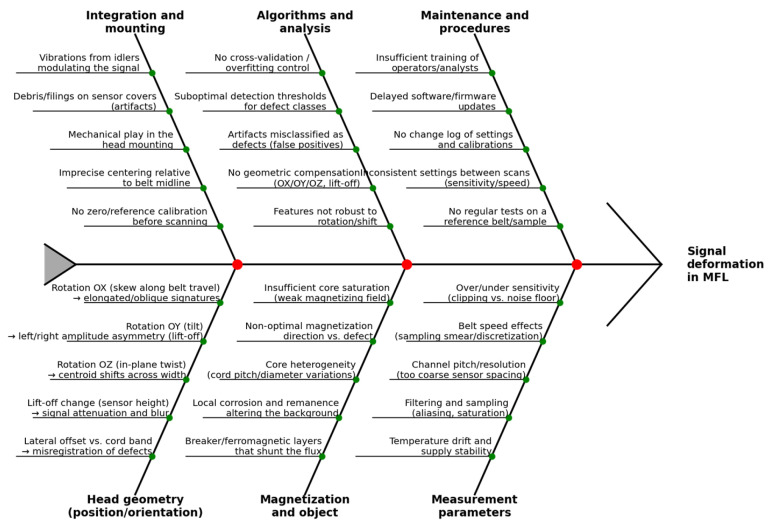
Ishikawa diagram of MFL signal error and uncertainty sources in multi-channel measurement systems.

The second Ishikawa diagram (Figure 4) organizes sources of MFL signal errors and uncertainty in multi-channel systems. It focuses on factors affecting measurement reliability and repeatability—from sensor head geometry (rotations OX/OY/OZ, lift-off, lateral shifts), through magnetization and object properties (saturation, magnetization direction, core heterogeneity), acquisition parameters (sensitivity, speed, channel resolution, filtering), to mechanical integration, analytical algorithms, and maintenance procedures. Each of these “branches” directly influences signal amplitude, shape, and centroid position, as well as the risk of misclassification (false positives/negatives).

The DiagBelt+ diagnostic system (ver. 1.1), developed and continuously improved at Wrocław University of Science and Technology, is based on the magnetic method. It produces two-dimensional field disturbance maps with a spatial resolution of 25 mm per channel, enabling defect inventorying and temporal tracking as well as degradation trend modeling for short-term forecasting and replacement planning [22]. Studies have shown that aggregated indicators (defect density/area per meter) and their growth rates can be modeled using regression approaches and applied for maintenance planning and belt replacement scheduling [15,18,23].

Laboratory and industrial tests using DiagBelt+ on belts with artificial core defects have confirmed the effectiveness of both statistical and machine learning methods for defect-type classification [24,25].

Despite the extensive literature on the influence of lift-off and magnetization parameters on MFL signals, the quantitative impact of sensor head orientation in multi-channel systems for ST belts (rotations about the belt-related axes) remains poorly recognized. However, studies of MFL in other objects have shown that changes in magnetization direction and probe tilt/twist can significantly alter signal sensitivity and shape [20,26,27].

The DiagBelt+ measurement head (BeltScan, Southport, QLD, Australia) uses two permanent magnet bars to generate a predominantly longitudinal magnetization of the steel cords. In an undamaged cord, the magnetic flux remains confined within the steel due to its high permeability. When a discontinuity is present (e.g., a cord cut, local cross-section loss, corrosion), the local permeability decreases, forcing part of the magnetic flux to escape into the surrounding medium in the form of a magnetic flux leakage (MFL) field.

The sensing coils embedded inside the sealed BeltScan measurement bar detect the temporal variation in this leakage field as the belt moves over the head. Although the detailed internal coil geometry is proprietary and not disclosed by the manufacturer, the physical principle follows established MFL theory: the amplitude, symmetry, and spatial distribution of the leakage field depend on the direction of magnetization, the defect structure, and the orientation of the sensor array.

Therefore, rotations of the head around OX, OY, and OZ alter the relative alignment between the magnetization field and the sensing coils, resulting in systematic geometric distortions of the recorded magnetic maps, which are quantified in this study.

This paper presents an experimental quantitative assessment of the influence of DiagBelt+ head orientation on the magnetic signal recorded above artificial steel-core defects under controlled laboratory conditions (varied belt speeds and sensitivity thresholds). Rotations around the OX (skew along belt motion), OY (tilt—causing asymmetric distance to covers), and OZ (in-plane twist) axes were analyzed, and the resulting signals were parametrized and compared.

Although the general influence of probe tilt and coil orientation on MFL signals has been discussed in previous studies, these works typically focus on single-probe configurations or localized effects. In contrast, the present study considers a multi-channel MFL system applied to an extended object with a complex magnetic field geometry, where the entire sensor array may experience spatial misalignment.

To the best of our knowledge, the quantitative impact of full-array rotation (OX, OY, OZ) on the geometry of recorded magnetic maps has not been systematically evaluated. This constitutes the specific research gap addressed in this work.

## 2. Materials and Methods

### 2.1. Test Stand

**Figure 5:**
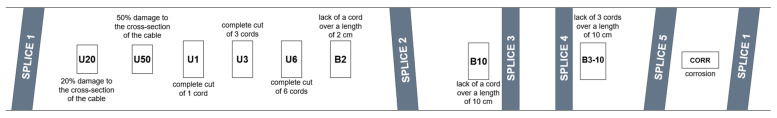
Layout of artificial defects and belt splices in the tested conveyor belt.

The measurements were carried out on a belt conveyor located in the Belt Transport Laboratory at Wrocław University of Science and Technology. The conveyor is equipped with a variable-speed drive, allowing operation in the range of 0.1–7 m/s. The test object was a steel-cord conveyor belt containing artificially modeled core defects. The belt was 400 mm wide, with a total length of 17,400 mm, and a core strength class of ST2500. The steel cords had a diameter of 5.6 mm and a pitch of 15 mm. As in all ST-class steel-cord belts, the cords are made of ferromagnetic wire with high magnetic permeability, ensuring effective magnetisation in MFL-based diagnostic systems. The individual belt sections were connected with five vulcanized splices. The layout of the defects and splices is shown in Figure 5.

**Figure 6:**
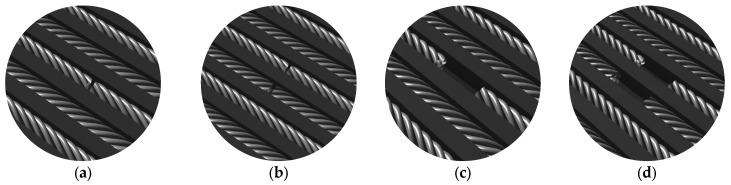
Schematic representation of artificial defects in the steel cords: (**a**) single cord cut, (**b**) two adjacent cords cut, (**c**) missing cord over 2 cm length, (**d**) two adjacent cords missing over 2 cm length.

**Figure 7:**
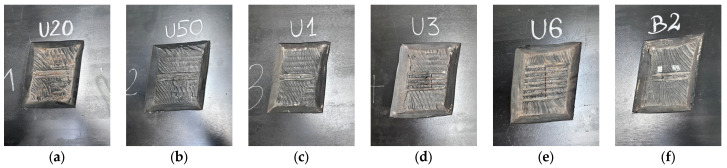
Actual appearance of artificial defects on the belt surface: (**a**) 20% cord cross-section loss, (**b**) 50% cord cross-section loss, (**c**) single cord cut, (**d**) three adjacent cords cut, (**e**) six adjacent cords cut, (**f**) missing cord over 2 cm length.

A schematic model and actual appearance of the artificial defects are presented in Figure 6 and Figure 7, respectively.

The DiagBelt+ diagnostic system was used for defect signal acquisition. The operating principle and measurement capabilities of this system have been described in detail in previous studies [8,15,18]. The measuring head has a working length of 2250 mm, equipped with coils spaced 25 mm apart, which in the baseline configuration (without rotation) yields 16 measurement channels. The measurement head used in this study is a commercial multi-channel sensor manufactured by BeltScan (Australia). The sensing coils are embedded inside the sealed measurement bar and are not directly accessible. Therefore, detailed coil parameters such as winding diameter, number of turns, or inductance are proprietary and not disclosed by the manufacturer.

However, the geometric sampling characteristics of the array are known: the coils are positioned at fixed 25 mm intervals along the head. This channel spacing determines the effective coverage area and forms the basis for the spatial resolution of the magnetic flux leakage maps.

**Figure 8:**
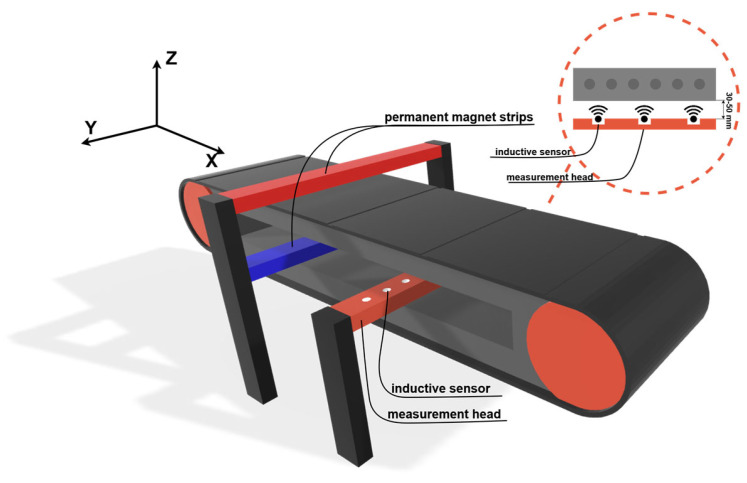
Schematic diagram of the DiagBelt+ measurement system installed on the conveyor belt.

The magnetic circuit of the measuring system consists of two permanent magnet bars mounted at a distance of 35 mm from the carrying and running covers of the belt. These bars provide quasi-static core magnetization, allowing the coils to record a two-dimensional magnetic flux leakage map as the belt passes over the measuring head. The lift-off distance between the head and the belt was measured at three points (left–center–right) before each configuration with an accuracy of 0.1 mm. Sampling was performed at 400 Hz, recording full loops of belt passage. Sensitivity levels were selected empirically based on prior experience. The schematic layout of the measurement system is shown in Figure 8.

Figure 8 illustrates the arrangement of the measurement head and the permanent magnets, together with the Cartesian coordinate system (X, Y, Z) used throughout this study. The X-axis corresponds to the belt travel direction, the Y-axis to the transverse direction across the belt width, and the Z-axis is perpendicular to the belt surface. The three rotations analyzed in this work are defined with respect to this coordinate system. The same convention is applied consistently in all further figures and descriptions.

**Figure 9:**
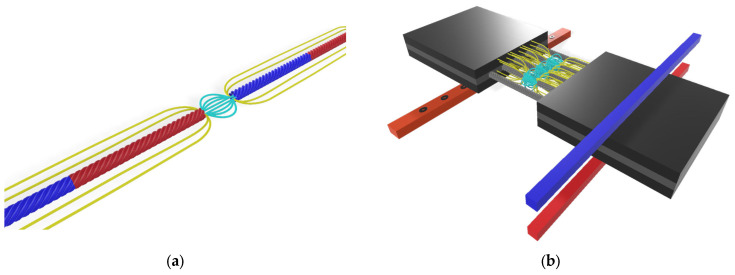
Conceptual magnetic field distribution around a magnetized steel cord and the formation of magnetic flux leakage (MFL) at a discontinuity. Yellow lines indicate the primary magnetization field, while cyan lines represent the leakage flux emerging from the defect (**a**) a single cord, (**b**) multiple cords.

To improve clarity, Figure 9 illustrates the approximate magnetic field distribution around a magnetized steel cord and the formation of the leakage field at a defect. The yellow lines represent the primary flux guided along the cord, while the cyan lines illustrate the leakage flux emerging at the discontinuity, which is detected by the sensing coils. The diagram is conceptual and intended to support understanding of the physical mechanism behind MFL-based defect detection.

**Figure 10:**
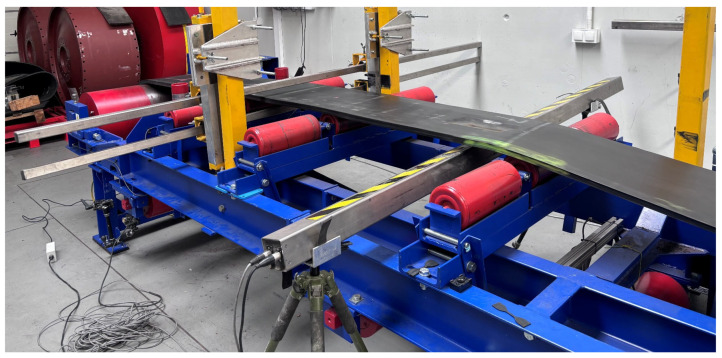
Conveyor test stand at the Belt Transport Laboratory.

A photograph of the test setup with installed permanent magnets and the measuring head is shown in Figure 10.

### 2.2. Definition of Rotational Axes and Head Orientation

To ensure an unambiguous description of configurations, a belt-fixed coordinate system was adopted:

- OX—Along the belt travel direction;
- OY—Across the belt width;
- OZ—Perpendicular to the belt plane (upward).

Three elementary rotations of the measuring head were analyzed, each varied individually (one-factor-at-a-time), while keeping the other two axes unchanged:

- Rotation about OX (skew): The measuring head is rotated within the belt plane around the X-axis, which is parallel to the direction of belt travel. As a result, one end of the head is shifted forward while the opposite end is shifted backwards relative to the belt motion, creating an in-plane skew without changing the lift-off distance.
- Rotation about OY (tilt): The head is rotated around the transverse axis (across the belt width). This results in different lift-off distances on the left and right sides of the sensor head.
- Rotation about OZ (in-plane twist): The head is rotated within the belt plane around the vertical axis. This produces an azimuthal rotation of the sensor array relative to the belt width direction.

**Figure 11:**
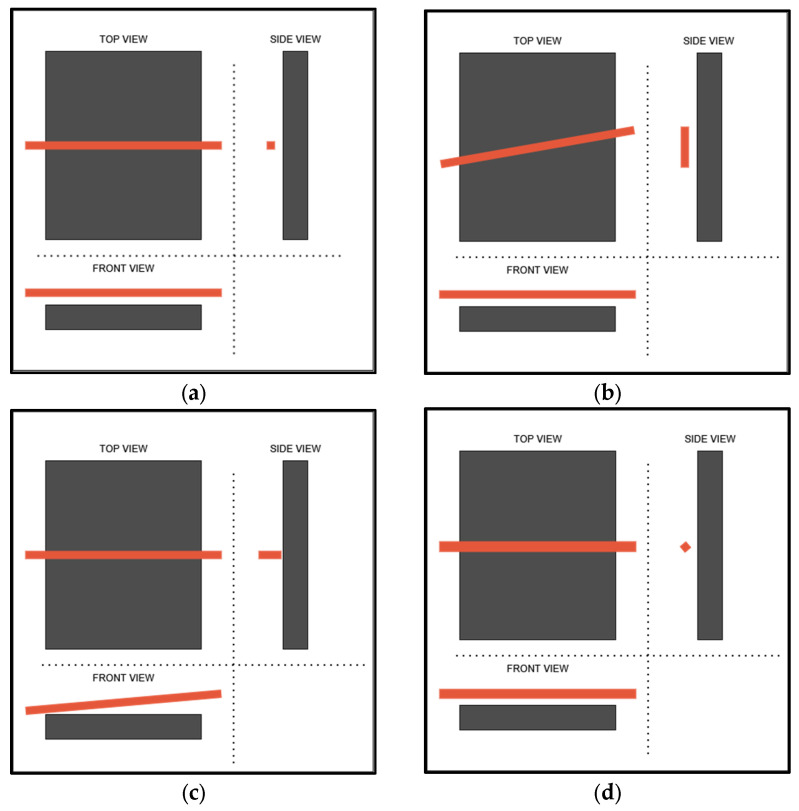
Schematic arrangement of the measuring head (orange) relative to the conveyor belt (gray): (**a**) baseline—no rotation, (**b**) rotation about OX, (**c**) rotation about OY, (**d**) rotation about OZ.

For clarity, Figure 11 presents schematic views of all three rotations (OX, OY, and OZ) together with the baseline configuration (no rotation).

**Figure 12:**
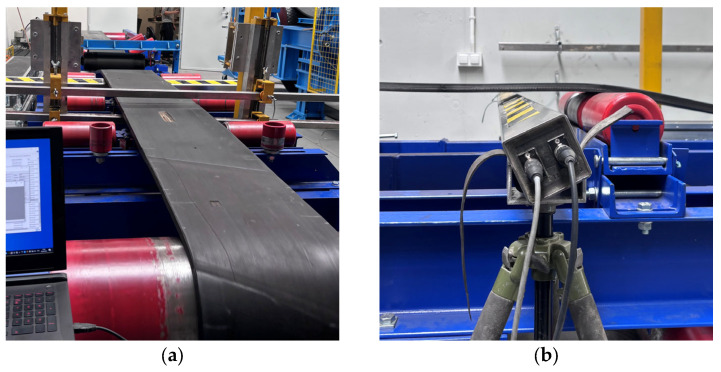
Measuring head rotation during testing: (**a**) rotation about OX, (**b**) rotation about OZ.

Photographs taken during the measurements showing rotations about the OX and OZ axes are presented in Figure 12.

### 2.3. Measurement Procedure and Data Processing

The experiments were performed at two belt speeds: 2 m/s and 4 m/s. For each speed, measurements were carried out at three sensitivity thresholds:

- For 2 m/s: thresholds = 2, 4, and 6;
- For 4 m/s: thresholds = 4, 6, and 8.

These values were selected based on the authors’ prior experience with magnetic measurements. The thresholds used in the experiments correspond to internal sensitivity levels of the commercial BeltScan (Australia) measurement system. They are dimensionless numerical settings that define the minimum signal amplitude required for pixel activation during binary segmentation. As they refer to the internal calibration of the device, they do not correspond to an external physical unit (e.g., mV or mT), but reflect relative detection sensitivity.

The range of head rotations was as follows:

- OX: 0°, 10°, 20°, and 30°;
- OY: 0° and 26.6° (corresponding to lift-off differences of 30–30 mm and 20–40 mm, respectively);
- OZ: 0°, 13°, and 24° (limited by the available stand geometry).

By varying only one rotation at a time relative to the baseline position, seven rotation sets were obtained. Considering 2 belt speeds and 3 sensitivity thresholds for each speed, this resulted in 42 measurement configurations, with 10 full belt loops recorded for each configuration.

Signals were recorded as 2D matrices (X-axis: belt travel direction; Y-axis: channel index/transverse position). Each scan loop produced one data matrix exported to a CSV file with dimensions [number_of_channels] × [number_of_samples]. Metadata were encoded in the file name as rotX_rotY_rotZ_v_s.

The sampling frequency was 400 Hz, and the acquisition time per loop was 8.7 s at 2 m/s and 4.35 s at 4 m/s, corresponding to spatial resolutions of 5 mm and 10 mm, respectively. Although the physical width of a single cord cut is typically below 5 mm, the magnetic flux leakage (MFL) generated at a discontinuity extends over a much larger region. Due to the strong permeability contrast, the leakage flux spreads around the defect, and its effective action range exceeds the physical dimensions of the damage, often covering 30–60 mm depending on magnetization conditions. Therefore, the spatial resolutions of 5 mm and 10 mm used in this study are sufficient to capture the full magnetic signature of both small defects and larger ones present in the benchmark belt.

Subsequent signal analysis was performed in Python (ver. 3.11) using the following libraries: NumPy (ver. 2.2.6), SciPy (ver. 1.15.2), scikit-image (ver. 0.52.2), pandas (ver. 2.2.3), and matplotlib (3.10.3). The analysis code included modules for CSV import and validation, normalization and filtering, signal segmentation, feature extraction, and tabular aggregation of results.

Repeatability was evaluated using the coefficient of variation (CV) of extracted features across k loops per configuration, while system stability was verified by periodic reference scans and Bland–Altman comparisons against the baseline (0°, 0°, 0°).

The consolidated results from all loops and configurations were stored in an Excel database wyniki_chmury2_clean.xlsx. Each row corresponded to one scan loop and included:

- Acquisition and geometric metadata (axis, angle_deg, v_ms, sensitivity_mV, lift_off_mm, loop_id, timestamp);
- Defect labels (defect_id, defect_class, x_position_mm, cord_index);
- Signal features describing the size, position, and shape of magnetic flux leakage regions, such as area, length, width, centroid coordinates, shape moments, compactness, and regularity indicators.

All values were converted from pixel to physical units (mm, mm^2^) using known belt speed, sampling frequency, and channel spacing.

Table 1 summarizes the set of geometric and shape features automatically extracted for each scan loop, including both primary dimensions and morphology descriptors computed from geometric moments and contour properties.

The obtained feature set was used to assess the influence of the DiagBelt+ head orientation on the spatial distortion and statistical characteristics of recorded magnetic field maps.

## 3. Results

### 3.1. Reference Measurement (0°, 0°, 0°)

**Figure 13:**
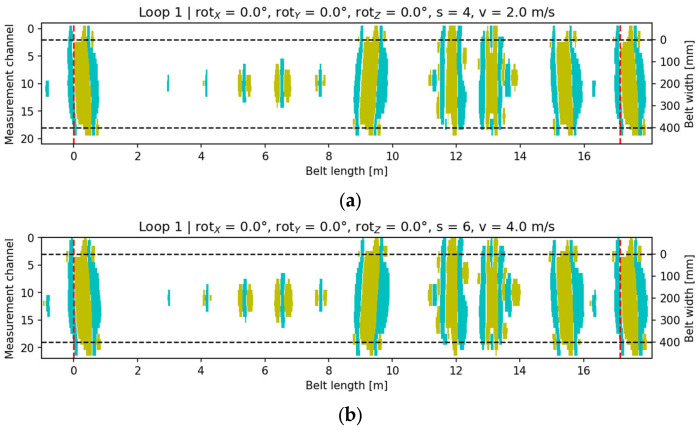
Visualization of the recorded signal for the baseline configuration: (**a**) v = 2 m/s, s = 4; (**b**) v = 4 m/s, s = 6. Cyan indicates the core-damage signal (pixels with a value of +1), yellow denotes accompanying signals (pixels with a value of −1), and the black dashed lines mark the conveyor belt width.

In the reference measurement with the head orientation (0°, 0°, 0°), a stable and symmetric magnetic field distribution was obtained over the area of artificial core defects. The signal map (Figure 13) shows the typical pattern of bands parallel to the belt travel direction, with no visible geometric distortions. A distinct amplitude maximum was observed in the central zone corresponding to the defect, with symmetric distributions along the left and right edges. The signal energy centroid was located close to the geometric center of the defect, and the measured length and width parameters were consistent with typical DiagBelt+ laboratory observations. This dataset was adopted as the baseline reference for subsequent rotational comparisons.

### 3.2. Effect of Rotation Around OX Axis

**Figure 14:**
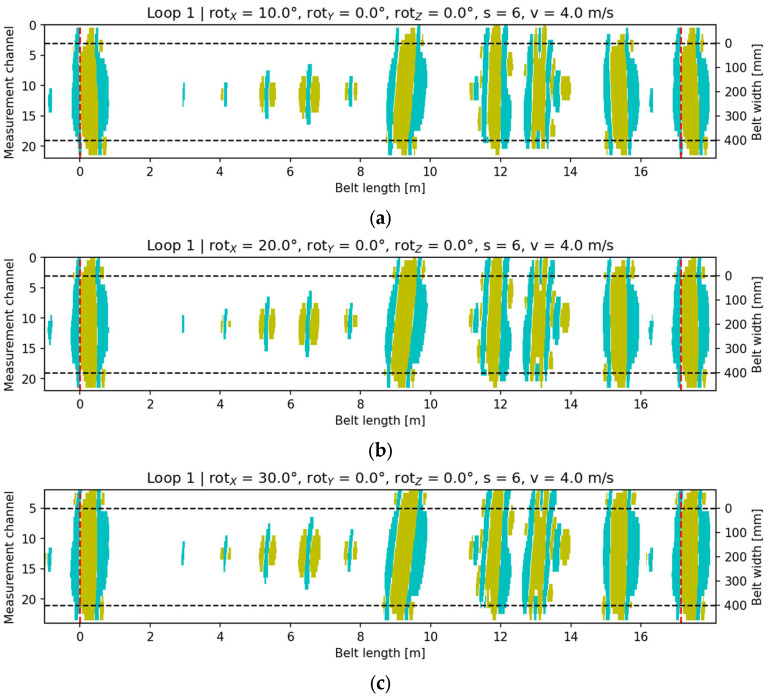
Signal visualization for configuration rotY = 0°, rotZ = 0°, v = 4 m/s, s = 6: (**a**) rotX = 10; (**b**) rotX = 20; (**c**) rotX = 30. Cyan indicates the core-damage signal (pixels with a value of +1), yellow denotes accompanying signals (pixels with a value of −1), and the black dashed lines mark the conveyor belt width.

Rotation of the head around the OX axis (skew along the belt motion direction) produced characteristic elongation and an oblique inclination of the signal bands in the 2D maps. As the rotation angle increased to 30°, a systematic shift in the signal centroid along the X-axis and a change in the main ellipse orientation were observed. Visualizations (Figure 14) show that even at 10°, a distinct deviation of the signal axis from parallel alignment with the belt direction appears.

**Figure 15:**
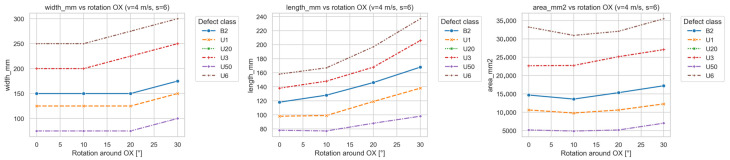

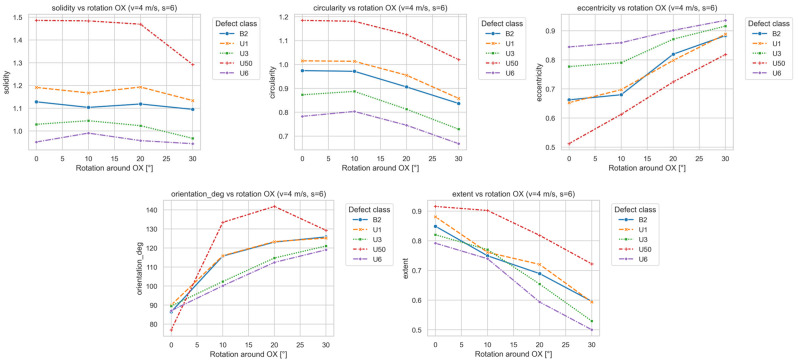
Dependence of signal parameters on OX head rotation.

As the rotation angle increased, the analyzed metrics exhibited systematic trends indicating geometric distortion of the signal while maintaining nearly constant total magnetic energy. This confirms that the rotation primarily modifies the shape, orientation, and compactness of the signal rather than its amplitude. Figure 15 illustrates how the signal parameters depend on the OX head rotation.

Quantitative analysis revealed a quasi-linear increase in the orientation_deg parameter, describing the inclination of the signal band relative to the belt direction. For most defect types (especially U1, U3, and U6), orientation increased by approximately 30–40° for head rotations up to 30°, clearly confirming the influence of head alignment on the directional representation of the signal.

A similar trend was observed for eccentricity, which increased systematically with the rotation angle, indicating elongation of the signal region along the belt direction. This effect was most pronounced for multi-cord defects (U3, U6), where eccentricity rose by more than 20% relative to the reference measurement—interpreted as apparent “stretching” of the signal caused by the altered sensor trajectory over the defect. In automated analysis, such geometric distortion may be misinterpreted as a larger defect extent.

The parameters length_mm and width_mm, describing the region’s longitudinal and transverse span, also increased with rotation, though the change in length was more pronounced. This indicates that the same defect was recorded over a longer sampling interval due to skew-induced sensor path displacement. The width increased moderately (≈15%) owing to contour blurring effects.

In contrast, parameters describing the total size and compactness—area_mm2, solidity, circularity—showed much lower sensitivity to rotation. The total signal area remained relatively stable (differences < 10%), confirming the purely geometric nature of the effect. Nevertheless, solidity and circularity decreased gradually with angle, especially for large defects (U6), indicating a less compact and more irregular contour.

### 3.3. Effect of Rotation Around OY Axis

Tilting the head around the OY axis resulted in amplitude asymmetry between the left and right sides of the signal map.

**Figure 16:**
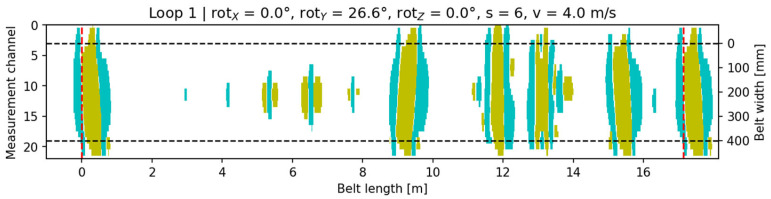
Signal visualization for configuration rotX = 0°, rotY = 26.6°, rotZ = 0°, v = 4 m/s, s = 6. Cyan indicates the core-damage signal (pixels with a value of +1), yellow denotes accompanying signals (pixels with a value of −1), and the black dashed lines mark the conveyor belt width.

As the tilt angle increased, the side with greater lift-off exhibited reduced signal amplitude and a shift in the centroid toward the opposite side (Figure 16).

**Figure 17:**
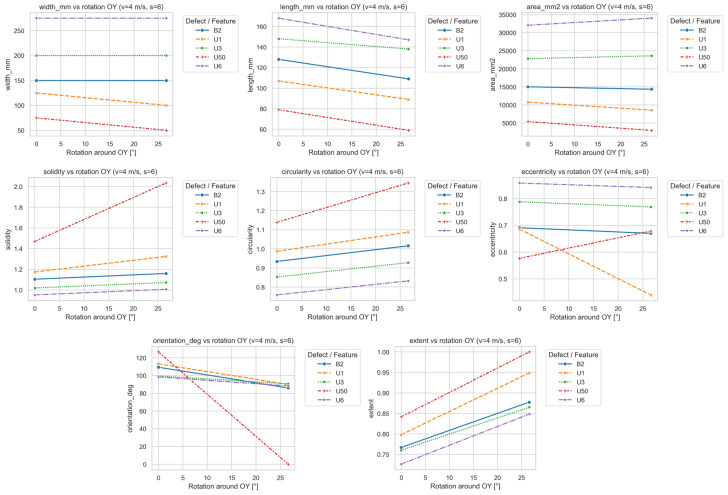
Dependence of signal parameters on OY head rotation.

Figure 17 illustrates how the signal parameters depend on the OY head rotation.

Rotation about OY introduced pronounced asymmetry in the recorded magnetic field distribution. Increasing tilt produced a difference in signal amplitude between the left and right sides of the head due to lift-off asymmetry. This effect altered the geometric parameters of the reconstructed defect image, exhibiting behavior distinct from that of OX rotation.

The most evident change was observed for orientation_deg, which, in contrast to OX rotation, decreased with increasing OY angle—indicating apparent “straightening” of the signal caused by the relative shift in the detection center toward sensors closer to the belt. This was especially pronounced for defect U50, where orientation changed by more than 100° between 0° and 25° tilt. For other defects, the decrease was moderate, suggesting that signal directionality is less sensitive to OY than to OX rotation.

Eccentricity exhibited mixed behavior: for some defects (e.g., U1), it decreased, implying a more compact shape, whereas for others (U50), it increased. This confirms that tilt does not affect all defect types in the same way; the response depends on defect morphology and its lateral position.

The length_mm parameter decreased with tilt angle, while width_mm remained nearly constant, indicating that the detector recorded the signal for a shorter duration due to attenuation on the lifted side. For large defects (U3, U6), length reduction reached 15–20% at maximum tilt.

Morphological parameters (circularity, solidity, and extent) increased with tilt, indicating a more regular and filled signal region. This can be attributed to reduced contrast between sensor channels—lower amplitude areas blend into the background, producing smoother contours. This trend was strongest for U50 and U1, suggesting that lower-amplitude signals are more susceptible to local blurring caused by uneven lift-off.

The area_mm2 parameter remained nearly constant, confirming that OY tilt affects spatial distribution and shape rather than total magnetic energy. Defects U1 and U50 were the most sensitive to tilt, while U3 and U6 showed greater shape stability.

### 3.4. Effect of Rotation Around OZ Axis

**Figure 18:**
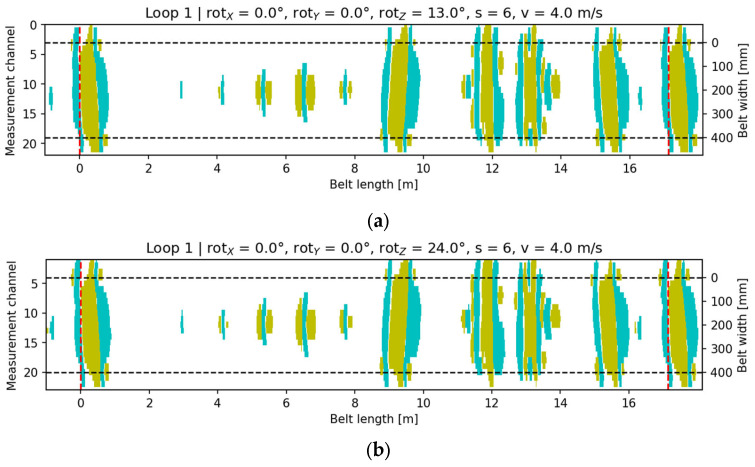
Signal visualization for configuration rotX = 0°, rotY = 0°, v = 4 m/s, s = 6: (**a**) rotZ = 13°; (**b**) rotZ = 24°. Cyan indicates the core-damage signal (pixels with a value of +1), yellow denotes accompanying signals (pixels with a value of −1), and the black dashed lines mark the conveyor belt width.

Rotation in the belt plane (around OZ) caused a diagonal twist of the signal map across the belt width. A clear shift in the signal centroid (center_col) and increased eccentricity were observed, indicating diagonal stretching of the signal region (Figure 18).

**Figure 19:**
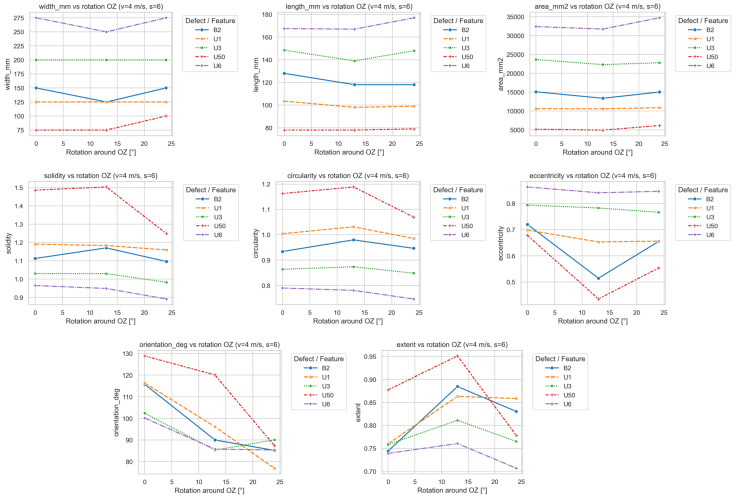
Dependence of signal parameters on OZ head rotation.

Figure 19 illustrates how the signal parameters depend on the OZ head rotation.

Rotation around OZ caused characteristic displacements and geometric distortions of the magnetic signal. Unlike OX and OY, this effect results not from lift-off differences but from angular misalignment of the entire sensor array relative to the vertical axis, altering the orientation of the magnetic field lines.

The most significant changes occurred in orientation_deg, which decreased systematically with increasing rotation angle, meaning that the signal’s major axis became more parallel to the belt direction. For most defects (U1, B2, U50), the decrease was nearly linear (≈20–30° within 0–25°). This can be interpreted as a rotation of the sensor array relative to the defect axis, causing the signal map to be “twisted” in the transverse plane.

The eccentricity parameter showed non-monotonic trends: some defects (U50, B2) exhibited local minima near 10–15°, suggesting partial compensation of orientation errors at small twists. At larger angles (>20°), eccentricity increased again. For major defects (U3, U6), the effect was smaller, indicating greater magnetic-field stability.

The parameters length_mm and width_mm remained within a narrow range, with slight minima near 10–15°, consistent with temporary compensation effects.

Morphological indicators (circularity, solidity, extent) increased up to 15°, suggesting more compact and regular shapes, then decreased again for higher angles, indicating dispersion and irregularity. The most pronounced variation occurred for defect U50, with local maxima of circularity and extent at 10–15°.

The area_mm2 parameter remained stable (fluctuations ≤ 10%), confirming the geometric rather than energetic nature of these effects. Larger defects (U3, U6) showed greater robustness to OZ rotation, while smaller, weaker signals (U1, U50) were more sensitive.

### 3.5. Quantitative Comparison

Rotation around OX caused systematic geometric distorsion of the signal pattern—an increase in orientation_deg and eccentricity, and a decrease in circularity and extent, with nearly constant area_mm2. These distortions intensified with both rotation angle and defect size. Thus, even slight skew (5–10°) may result in significant misinterpretation of defect geometry in automated diagnostic systems. It is therefore recommended to implement geometric compensation, e.g., through orientation correction or affine transformation alignment between scans.

Rotation about OY primarily introduced amplitude asymmetry and apparent shape modification rather than spatial elongation. With increasing tilt, orientation, and length decreased, while solidity, circularity, and extent increased, reflecting lift-off asymmetry between head sides. To mitigate this, automatic cross-channel sensitivity compensation and periodic alignment verification are recommended in multi-channel MFL systems.

Rotation around OZ produced moderate but distinct geometric distortions, mainly affecting orientation and centroid position. Small twists (≈10–15°) improved compactness, while larger ones degraded regularity and symmetry. Although less severe than OX or OY effects, OZ rotation should be considered in sensor calibration, particularly when position-based defect localization is critical.

## 4. Discussion

The results clearly demonstrate that measurement head rotation relative to the conveyor belt is a major source of geometric distortion in MFL (Magnetic Flux Leakage) diagnostic systems. In the DiagBelt+ setup, such distortions arise from magnetic field asymmetry and nonlinear sensor response to angular and distance variations.

For OX rotation (skew along the belt), the dominant factor is the phase shift between sensor channels. As the magnetic flux from a defect passes sequentially under each sensor, misalignment causes asynchronous detection, producing apparent elongation and skew in the reconstructed map. The observed increases in orientation_deg and eccentricity confirm that at larger rotation angles, sensors record the defect over an extended time window without an amplitude change.

For OY rotation (tilt), the effect is mainly amplitude asymmetry due to lift-off differences. Even a few millimeters of variation significantly attenuates the signal on the lifted side, distorting the field map and shortening the apparent signal. This explains the observed decrease in orientation and length with a concurrent increase in solidity and circularity. Similar effects are well documented in the MFL literature for inductive sensors, where lift-off variations cause misrepresentation of defect shape and location.

For OZ rotation (in-plane twist), distortions stem from the altered alignment between magnetic field lines and the sensor array. Small rotations (≈10–15°) improve symmetry and compactness, but larger ones (>15°) lead to centroid displacement and reduced regularity—potentially resulting in misclassified defect positions in automated analysis.

Practically, even minor head rotations (5–10°) can significantly affect diagnostic interpretation. In online systems such as DiagBelt+, improper mounting or frame misalignment may cause systematic classification errors, especially in long-term industrial installations where vibration, wear, or mechanical deformation occur.

Potential countermeasures include:

- Geometric calibration through periodic reference-belt scanning and alignment to a geometric template;
- Automatic rotation compensation algorithms that correct signal orientation based on extracted features (e.g., orientation_deg, skew_row);
- Integration of machine learning approaches for automatic recognition of deformation patterns and detection of misalignment errors.

Such methods could substantially enhance the reliability of industrial diagnostics where manual recalibration is impractical.

## 5. Conclusions

It is important to distinguish between defect detection and defect interpretation in multi-channel MFL systems. All defects investigated in this study were reliably detected under every rotation scenario of the sensor head, confirming that detection robustness is not affected by misalignment. However, the geometric description of the recorded MFL signal (orientation, eccentricity, centroid position, apparent length and width) becomes distorted when the head is rotated around the OX, OY, or OZ axes. The resulting errors, therefore, concern the interpretation of defect type, position, and size rather than the ability to detect the defect itself.

The scientific novelty of this work lies in the first quantitative assessment of how full-array sensor head misalignment affects the geometry of 2D magnetic maps in a multi-channel conveyor belt inspection system. Practically, the results highlight the need for geometric compensation methods to ensure correct defect classification under industrial operating conditions.

The conducted research enabled quantitative evaluation of the measurement head rotation effects on MFL signal geometry in the DiagBelt+ system.

The obtained results identified which rotation types are most critical to diagnostic accuracy:

1. OX rotation produces the most severe geometric distortions—systematic increase in orientation_deg and eccentricity, with apparent elongation of the defect along the belt motion direction.
2. OY rotation causes amplitude asymmetry and reduction in signal length, while increasing compactness indicators (solidity, circularity).
3. OZ rotation mainly alters signal orientation and symmetry; at higher angles, centroid displacement and reduced regularity occur.
4. The total signal area (area_mm2) remains stable across all rotations, confirming the geometric (not magnetic) nature of changes.
5. In practice, even a 5–10° mounting error can produce deviations in geometric parameters exceeding the classification thresholds used in automated defect detection.

Practical recommendations for MFL diagnostic systems include the following:

- Implement orientation calibration procedures;
- Monitor the mechanical stability of sensor mounts and alignment;
- Apply geometric compensation algorithms in analytical software;
- Explore machine learning methods for automated detection and correction of signal distortion.

Future work will focus on the implementation and validation of these compensation algorithms within the DiagBelt+ software environment, and on evaluating their impact on real-world defect classification performance under industrial operating conditions.

## Figures and Tables

**Table 1:** Description of signal features extracted from defect maps.

Symbol/Column Name	Description
area	Number of pixels assigned to the defect cloud (region with −1 values).
area_mm2	Physical area of the defect cloud.
length	Extent of the cloud along the belt travel direction.
width, width_mm	Width across the belt (number of active channels/physical width).
center_row, center_col, center_meter	Coordinates of the cloud centroid along X (rows) and Y (channels), and in meters from loop start.
equiv_diameter_mm	Diameter of a circle having the same area as the cloud.
eccentricity	Eccentricity of the fitted ellipse (0 = circular, → 1 = elongated).
orientation_deg	Angle of the major axis of the fitted ellipse with respect to the belt direction.
skew_row, skew_col	Skewness of pixel distribution along X and Y axes.
hull_area_mm2, hull_perimeter_mm	Area and perimeter of the convex hull enclosing the cloud.
solidity	Solidity = ratio of area to convex hull area; compactness measure.
circularity	Shape regularity, independent of size.
bbox_area_mm2	Area of the axis-aligned bounding box.
extent	Fraction of bounding-box area occupied by the defect region.

## Data Availability

The dataset is available on request from the authors.
